# The Development of Cutaneous Lesions in Tropically Adapted Beef Cattle Is Associated with Hypersensitive Immune Response to Buffalo Fly Antigens

**DOI:** 10.3390/ani13122011

**Published:** 2023-06-16

**Authors:** Muhammad Noman Naseem, Ali Raza, Muhammad Kamran, Rachel Allavena, Constantin Constantinoiu, Michael McGowan, Conny Turni, Ala E. Tabor, Peter James

**Affiliations:** 1 The University of Queensland, Queensland Alliance for Agriculture and Food Innovation, Centre for Animal Science, St. Lucia, QLD 4072, Australia; m.naseem@uq.edu.au (M.N.N.); k.muhammad@uq.edu.au (M.K.); c.turni1@uq.edu.au (C.T.); a.tabor@uq.edu.au (A.E.T.); 2 The University of Queensland, School of Veterinary Science, Gatton, QLD 4343, Australia; r.allavena@uq.edu.au (R.A.); m.mcgowan@uq.edu.au (M.M.); 3 James Cook University, College of Public Health, Medical & Veterinary Sciences, Townsville, QLD 4810, Australia; constantin.constantinoiu@jcu.edu.au; 4 The University of Queensland, School of Chemistry & Molecular Biosciences, St. Lucia, QLD, 4072, Australia

**Keywords:** *Haematobia*, Cattle skin lesions, *Stephanofilaria*, buffalo fly, hypersensitivity

## Abstract

**Simple Summary:**

Skin lesions in cattle associated with feeding by *Haematobia* fly species are a significant health issue in northern Australian cattle. These lesions have most commonly been attributed to the effects of *Stephanofilaria* nematodes vectored by the *Haematobia* spp. flies. However, the exact etiology of these lesions and reasons for the variation between the cattle in the severity of lesions was unclear. This study investigated the cutaneous responses to fly and nematode antigens in lesion-resistant and susceptible cattle, which indicated that differences in the hypersensitivity response to buffalo fly (*Haematobia irritans exigua*) antigens is a key factor underlying the variation amongst the cattle in susceptibility-to-lesion development. There was no association found between the intensity of fly infestation and lesion susceptibility. The findings from this study suggest that further characterization of the skin response could lead to the identification of the biomarkers for selecting cattle with an increased resistance to buffalo fly lesion development.

**Abstract:**

This study investigated the role of cattle immune responses in the pathogenesis of buffalo fly (*Haematobia irritans exigua*) (BF) lesions. Brangus steers phenotyped for lesion development were divided into three groups: high lesion susceptibility (HL), low lesion susceptibility (LL) and no lesions (NL), based on lesion severity scores. Each steer was injected intradermally with different concentrations of BF, *Onchocerca gibsoni* (Og), and *Musca domestica* (Md) antigens. At 1 h post-injection, wheal areas at BF injection sites were found to be significantly larger in HL than NL cattle, but there were no significant differences (*p* < 0.05) found between either the HL or NL cattle and LL cattle. At 24, 48, and 72 h post-injection, the skinfold thickness response to both BF and Md antigens was significantly greater in the HL group than the NL group. However, skin thickness was significantly greater for the BF antigens than the Md antigens (*p* < 0.05). There were no significant differences found between the LL and NL animals in response to the BF antigens at any time, and no significant differences were determined between any of the lesion groups in response to the Og antigens. Histological examination of skin sections taken from the BF antigen injection sites in HL cattle at 72 h post-injection revealed necrosis of the epidermis and superficial dermis, along with severe eosinophilic inflammation. This study suggests that differences in the hypersensitivity to BF antigens underlie differences amongst the cattle in their susceptibility to the development of BF lesions, and breeding for immune-related biomarkers may assist in selecting more BF lesion-resistant cattle.

## 1. Introduction

Flies from the genus *Haematobia* are small, obligate hematophagous ectoparasites that feed mainly on cattle and buffalo. Buffalo flies (BFs) (*Haematobia irritans exigua*) are a major pest of north Australian beef and dairy herds causing economic and welfare impacts [1]. They are widely spread through South and South East Asia and into Papua New Guinea, whereas their closely related horn flies (*Haematobia irritans irritans*) are spread through North and South America, Europe, and North Africa [2]. Each fly feeds on cattle blood up to 40 times daily, and fly counts can reach up to several thousand per animal [3]. Buffalo fly lesions are manifested most frequently as dermatitis or ulcerated areas close to the medial canthus of the eye and along the lateral and ventral neck and abdomen [4,5]. Clinical appearance can vary from raised, dry, alopecic, hyperkeratotic, or scabbed areas to severe hemorrhagic ulcerated lesions [6,7], with up to 95% of cattle herds affected in some areas of northern Australia [4]. Lesions can penetrate deeply into the dermis and cattle often scratch at the affected areas to relieve itching. This can cause permanent hide damage that reduces the hide value, restricts the marketability of the affected stock, and may reduce market value of the affected cattle by up to $0.35/kg in saleyards [1,5]. In addition, the presence of lesions is a significant animal welfare concern and can increase cattle susceptibility to other infections.

Variation in the severity of lesions amongst the cattle within herds is well known [5], and although the development of skin lesions is associated with BF feeding, there appears to be a poor correlation between the BF counts and lesion development [5,8]. A filarial nematode *Stephanofilaria* sp., which is vectored by the BF, has been found to be associated with the pathogenesis of BF lesions [7,9]. However, *Stephanofilaria* has not been detected in all BF lesions [10], suggesting that nematode infections might not be essential for BF lesion development. Naseem et al. [11] isolated identical strains of *Staphylococcus agnetis* and *Staphylococcus hyicus* from BFs and lesions and suggested that these bacteria may contribute to lesion pathogenesis. However, the exact etiology of the BF lesions was not clear.

Horn flies are also reported to be the vector of *Stephanofilaria stilesi*, which has been implicated in the development of skin lesions in cattle in North and South America [12,13]. However, Edwards et al. [14] reported udder lesions in US cattle, which they suggested developed in response to their hypersensitivity to HF bites. Horn fly-associated eosinophilic dermatitis in cattle has also been reported in Argentina and France [15,16].

Kerlin and Allingham [17] described the development of a type 1 hypersensitivity response to BF antigens and saliva in response to buffalo fly exposure, but they did not investigate the association of this response with the underlying variations in cattle susceptibility to lesion development. This study reports the differences in the cutaneous immune responses to BF and nematode antigens in lesion-resistant and susceptible cattle.

## 2. Material and Methods

### 2.1. Animals

**Figure 1:**
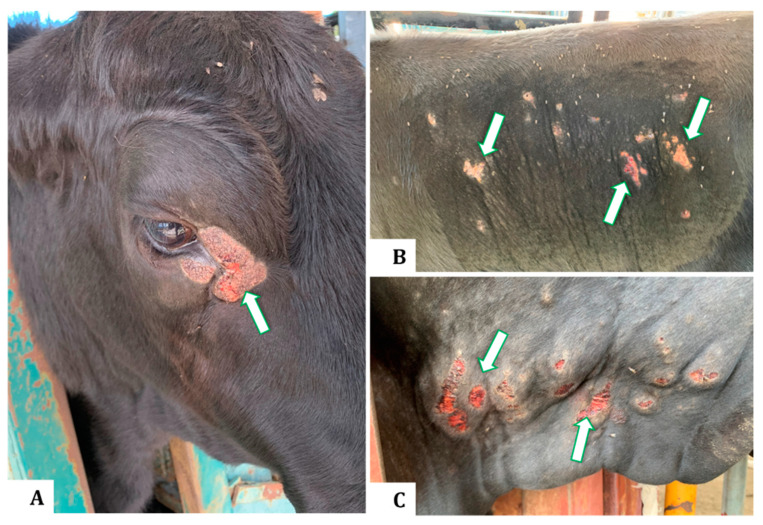
Clinical appearance of buffalo fly lesions in Brangus steers indicating raised, circumscribed, hairless, ulcerative, or scab-encrusted areas adjacent to the medial canthus of the eye (**A**), on the lateral abdomen (**B**), and the dewlap (**C**).

Brangus steers (*n* = 25) approximately three years of age kept at the University of Queensland Pinjarra Hills Research Precinct (−27.53, 152.91) were monitored quarterly over two years (2020 and 2021) for BF numbers and lesions (Figure 1). The dimensions of each lesion (length and breadth) were measured, and the area of each lesion was estimated as the area of an ellipse. There was a high degree of consistency in the rankings of these animals for buffalo fly lesion severity across the dates. Therefore, only lesion area estimates taken near the end of the second BF season (when lesion development was at its maximum) were used to classify animals for the study. On the basis of this assessment, these animals were divided into three groups, which were as follows: highly lesion-susceptible animals (HL) with multiple body lesions or eye lesions >15 cm^2^ in area (*n* = 7), an intermediate group of low-lesion animals (LL) that had only small eye lesions with an area of <15 cm^2^ (*n* = 8), and lesion-resistant animals (NL) which had no lesions (*n* = 10). These studies were conducted under the UQ Animal Ethics approval no. QAAFI/469/18.

To estimate BF numbers, the animals were held in a small paddock on nine dates during 2019 and 2020 prior to hypersensitivity testing and were photographed from one side using a Canon SX40 HS Powershot digital camera with a telephoto lens. Numbers of BFs were later estimated from the images through visualization and counting using Image J software (v1.5a, Wayne Rasband, National Institute of Health, Bethesda, MD, USA), and an average fly count over the nine dates was calculated for each animal.

### 2.2. Preparation of Soluble Antigens of Buffalo Flies and House Flies

Buffalo flies and house flies (*Musca domestica*) (Md) were acquired from laboratory-grown colonies kept at the EcoScience Precinct (Dutton Park, QLD, Australia). These colonies were derived from locally caught BFs and house flies. House flies are closely related to buffalo flies, and Md antigens were included to control for the generic cross-reacting antigens and enable the identification of BF-specific reactions. The flies were kept on the bench for 24 h at room temperature to clear the ingested blood out of the gut, immobilized by chilling at 4 °C for 5 min, and then soluble antigens were prepared using minor modifications of the methods used in previous studies [17,18,19]. Briefly, for protein extraction, a protein extraction buffer solution was prepared by mixing Halt^TM^ protease and phosphatase inhibitor 100× (Cat. no. 1860932, Thermo Scientific, Waltham, MA, USA) in T-PER^TM^ tissue protein extraction buffer (Cat. no. 78510, Thermo Scientific, Waltham, MA, USA) in a 1:99 ratio. The flies (50 mg in weight) were placed in 2mL low protein-binding tubes with 750 µL of protein extraction buffer, followed by lysis in a tissue lyzer (TissueLyser II, QIAGEN Pty Ltd., Hilden, Germany) with 2 mm steel beads at 30 Hz/s for 2 min. These homogenates were then sonicated for 2 min at 40 MHz to produce a crude antigen. This antigen was then centrifuged (Eppendorf Centrifuge 5430R, Hamburg, Germany) at 20,817× *g* for 90 min at 4 °C. The supernatant was decanted and filtered through a 0.22 µm filter (Cat. no. SLGP033RS, Merck Millipore, Burlington, MA, USA) into a new 2mL low protein-binding tube. The filtered supernatant was then concentrated at 45 °C for 90 min using a concentrator (Eppendorf Concentrator Plus, Hamburg, Germany). Each pellet was then resuspended in 100 µL of phosphate buffer saline (PBS). The protein concentration was quantified with a Qubit^TM^ 4 fluorometer (Thermo Scientific, Waltham, MA, USA) using a Qubit^TM^ protein assay kit (Cat. no. Q33211, Invitrogen, Waltham, MA, USA).

### 2.3. Preparation of Onchocerca gibsoni Antigens

It was initially intended to use *Stephanofilaria* sp. to determine whether an inflammatory response to nematode antigens could be involved in the development of the skin lesions. However, due to COVID-19-related travel and access restrictions, it was not possible to source sufficient amounts of *Stephanofilaria* sp. antigens. Cross-reactivity amongst antigens from the filariid nematode species have been reported, and soluble antigens of the filarial nematodes of animals have previously been used to diagnose filariasis in humans [20,21]. Therefore, in the absence of sufficient quantities of *Stephanofilaria* sp. antigens, we used soluble antigens from *Onchocerca gibsoni* (Og), a closely related nematode parasite of cattle. Prior to the commencement of the study, steers were assessed for infection with *O. gibsoni* using a commercially available diagnostic test kit (TropBio Og4C3 Filariasis Antigen ELISA kit (Cat. no. KF1, CeLLabs, Sydney, NSW, Australia) and all returned negative results.

For Og antigen preparation, infected nodules were collected from freshly slaughtered cattle from an abattoir in North Queensland (Stuart, QLD, Australia) and were stored at −20 °C until use. Male and female nematodes were isolated from the nodules by dissection and washed three times with sterile normal saline, and then antigens were prepared using the protocol described for the fly antigens.

### 2.4. Intradermal Injection of Soluble Antigens

Buffalo fly, house fly, and nematode antigens were diluted 10-fold from 1 to 0.01 mg/mL. An area of 30 × 30 cm was clipped from the left side of each steer, starting from the last 2–3 ribs under the transverse process of the vertebrae, and a grid was marked to define the injection sites. There were three rows of three sites in each clipped area (each injection site was 8 cm apart from the other). The clipped area was washed and disinfected (with 0.05% chlorhexidine aqueous solution) before injections. In the first row, 0.1 mL of BF antigens containing 100 (BF100), 10 (BF10), and 1 (BF01) µg of BF antigens, respectively, were injected intra-dermally using an insulin syringe and an 8 mm length, 30-gauge needle. In the second row, 0.1 mL of Og antigens containing 100 (Og100) and 10 (Og10) µg antigens was injected. In the third row, 0.1 mL of Md antigen with 100 (Md100) and 10 (Md10) µg antigens was injected at the first two sites, and 0.1 mL of PBS was injected at 3rd site as a negative control.

### 2.5. Measurement of the Skin Responses

Each steer was examined 1 h after injection, and the length and width of the skin reactions surrounding each injection site were subsequently measured. The wheal area was calculated as the area of an ellipse for analysis. Skinfold thickness was measured at four sites on each animal before injection, and then at each injection site after 24, 48, and 72 h following injection, respectively, using a digital caliper. To adjust for the initial variation between the animals in skin thickness, pre-injection skin thickness readings were subtracted from the post-injection skin-thickness measures, and the adjusted values were used for statistical analyzes.

### 2.6. Biopsy Collection and Processing

At 72 h post-injection, biopsies were collected from 100 ug BF antigen injection sites from five steers with the highest lesion scores obtained from the HL group. Samples were collected using 8 mm skin punches after applying ring local anesthesia with 3 mL of 2% lignocaine. One biopsy was also collected from a PBS injection site from each of the biopsied steers as a control. Biopsies were prepared by the routine method of 10% neutral-buffered, formalin-fixed, paraffin-embedded 4 μm sections stained with hematoxylin and eosin (H&E).

### 2.7. Statistical Analysis

Differences between the lesion groups in terms of BF numbers were analyzed by one-way analysis of variance on untransformed data. Differences in the wheal areas between the animals were analyzed by the two-way analysis of variance with the lesion score group and antigens as the main effects, whereas analyzes for skin thickness at 24, 48, and 72 h were performed with a repeated measures model. Treatment means were compared by Least Significant Differences (LSDs) calculated at *p* ≤ 0.05, and all analyzes were performed using Genstat^®^ v21 (VSN International, Hemel Hempstead, UK).

## 3. Results

### 3.1. Buffalo Fly Numbers in the Different Lesion Groups

**Figure 2:**
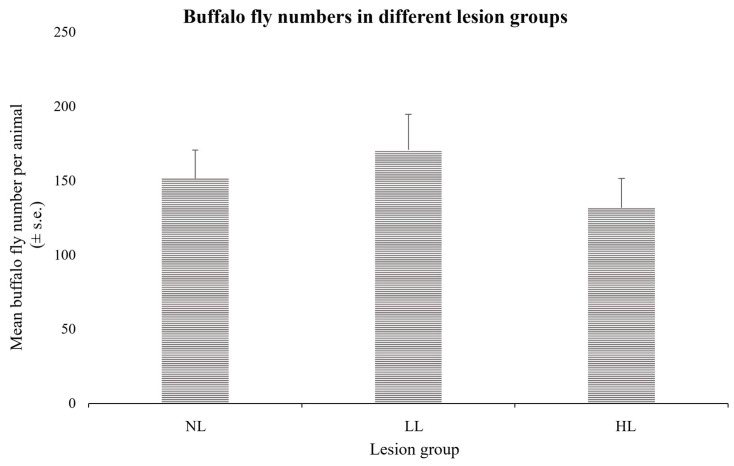
Mean buffalo fly numbers per animal (±standard error) over nine counts in cattle divided into the three lesion groups. Differences between the groups were not significant (*p* > 0.05).

Mean BF numbers (standard errors) for the three groups were 151.5 (±19.1), 170.7 (±24.0), and 131.8 (±19.7) for the LR, LL, and HL groups, respectively, and there was no significant differences observed between the BF numbers in the three groups (*p* > 0.05), indicating that the occurrence of the lesions was not simply a reflection of high BF numbers on a few cattle (Figure 2).

### 3.2. Skin Responses to Different Antigens

#### 3.2.1. Wheal Area at 1 h Post-Injection

**Figure 3:**
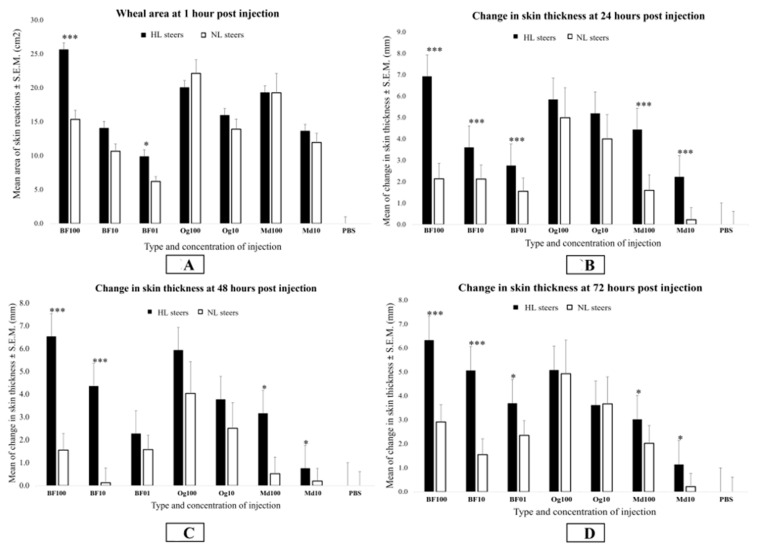
Bar graphs showing differences in the means for the wheal area (**A**) and changes in the skinfold thickness (**B**–**D**) between lesion-susceptible (HL) and -resistant (NL) steers in response to different concentrations of the buffalo fly, house fly, and nematode antigens at 1, 24, 48, and 72 h post-injection. Changes in the skin thickness were calculated by subtracting the skin thickness before injection from thickness at 24, 48, and 72 h post-injection. Different concentrations of buffalo fly (BF) antigens are indicated by BF100 (100 µg), BF10 (10 µg), and BF01 (1 µg) on the X-axis, respectively, whereas *Onchocerca gibsoni* (Og) and house fly (Md) antigens concentrations are indicated by Og100 (100 µg) and Og10 (10 µg), and Md100 (100 µg) and Md10 (10 µg), respectively. Significance of analysis of variance, * *p* < 0.05; *** *p* < 0.001.

At 1 h post-injection, inflammatory reactions in the form of characteristic wheal and flare reactions were found to have developed to all concentrations of BF, Md, and Og antigens in all animals. There were no inflammatory responses visible from PBS injections. The HL cattle had significantly larger wheal areas with 100 and 1 μg BF antigens than NL cattle (*p* < 0.05). Although the difference in wheal areas with the BF 10 μg concentration was not significant at the 5% level, the wheal areas were also larger in the HL than in the NL cattle, and this difference was found to be significant at *p* < 0.1, suggesting that the lack of significance was a reflection of the relatively low numbers of animals in the study rather than a real effect. When the responses of the LL and NL cattle were compared, there were no significant differences found with any of the antigens tested at any time point. When the responses of the HL cattle were compared to the responses of the LL cattle, the BF100 antigen wheal area was significantly larger in the HL cattle at 1 h (*p* < 0.05), but not at the later times. With the Md and Og antigens, there were no significant differences (*p* > 0.05) observed in the size of the wheal area between the lesion susceptibility groups at any time point (Figure 3A).

#### 3.2.2. Skinfold Thickness Response

Skinfold thickness responses to all concentrations of the BF and Md antigens were found to be significantly greater in the HL cattle than the NL cattle at the three times points after one hour (*p* < 0.05) except for the BF 1 μg concentration at 48 h (Figure 3B–D). However, skinfold thickness responses to the BF antigens were found to be significantly greater than for the Md antigens at the equivalent concentration in the HL cattle in all cases (*p* < 0.01). Skinfold thickness was also greater in the HL cattle than in the LL cattle with the BF100 antigens at 24 h (*p* < 0.01) (Figure S1). There was no significant differences observed between the LL and NL groups to any of the antigens tested at any time (*p* > 0.05) (Figures S2–S4). Skinfold thickness data (raw) is provided in Table S1.

When the HL group and the LL groups were compared, there was a significant difference observed with the BF01 concentration at 72 h (*p* < 0.05), but the difference was not significant at 48 h with the BF01 concentration, or with the BF10 and BF100 concentrations at either 48 h or 72 h (*p* > 0.05) (Figures S5 and S6). There was no significant difference observed between the HL and LL groups with the Md or Og antigens at any time (*p* > 0.05).

### 3.3. Pathological Changes in the Skin Tissue of HL Animals

#### 3.3.1. Gross Changes

**Figure 4:**
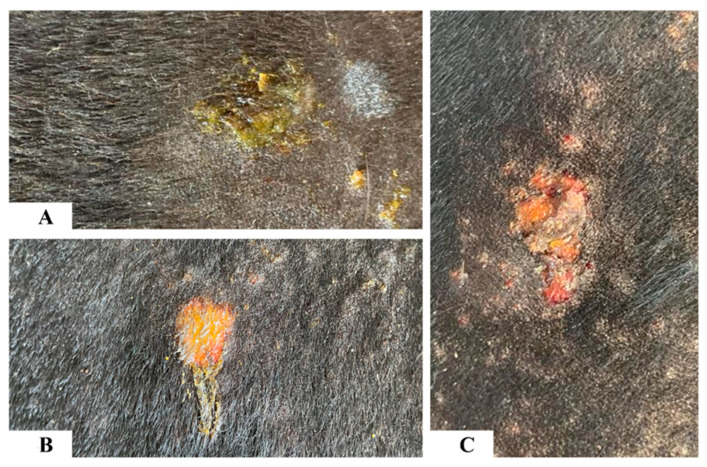
Photographs showing gross changes in the skin in response to buffalo fly antigens (100 µg) in high-lesion animals. At 24 h post-injection, there was a seepage of serous transudate drying on the surface of the buffalo fly injection site (**A**). At 48 and 72 h post-injection, the superficial epidermal layer was lost, and the buffalo fly injection site was covered with dry serous crust (**B**,**C**).

In HL animals one hour after the antigen injections, the area around the 100 µg BF antigen injection site was raised with redness and seepage of serous fluid at the injection site. At 24 h post-injection, further seepage was observed with serous transudate dried up on the surface of the BF antigen injection sites (100 µg and 10 µg) in the HL animals (Figure 4A). At 48 and 72 h after antigen injection, superficial epidermal disruption was noted at the 100 µg BF antigen injection site in the five most lesion-susceptible animals, and the development of a dry serous crust over the injection site was observed (Figure 4B,C). No cutaneous tissue damage was observed grossly in the LL and NL animals to any concentration of BF antigens at any time point, but skinfold thickness was found to have increased. Similarly, no gross skin damage was observed in any of the lesion animal groups in response to the Og and Md antigens.

#### 3.3.2. Histological Changes

**Figure 5:**
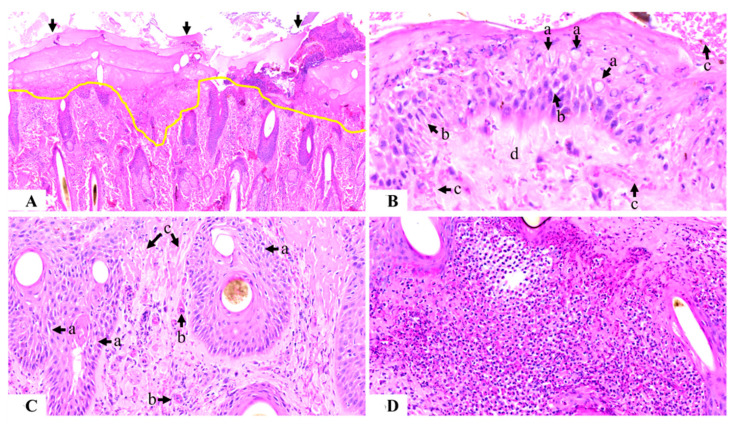
Photomicrograph of the histology of skin sensitized with buffalo fly antigens (100 µg): Figure (**A**) shows complete epidermal necrosis with ulceration extending to the superficial dermis (area above the yellow line) and covered with a thick serocellular crust (arrowed) (100×); Figure (**B**) shows partially damaged epidermis with ballooning degeneration (a), spongiosis (b), epidermal and sub-epidermal hemorrhage (c), and sub-epidermal collagenolysis (d) (400×); Figure (**C**) shows dermal damage, including spongiosis of the hair follicle (a), endothelial activity (b), and collagen fragmentation (c) (200×); and Figure (**D**) shows the loss of dermal connective tissue along with severe inflammation comprising neutrophils and eosinophils (200×).

There was acute partial-to-complete epidermal necrosis observed in all biopsied HL animals, with ulceration extending to the superficial dermis and involving the hair follicles (Figure 5A). In partially damaged epidermal layers, there was marked spongiosis, intracellular edema, and ballooning degeneration (Figure 5B). The affected area was covered superficially with a serocellular crust of varying thicknesses ranging from 400–500 µm, with cellular composition involving necrotic epidermal cells, neutrophils, and eosinophils.

The dermis was moderate to severely hemorrhagic, and collagen in the superficial dermal layer was fragmented, indicating collagenolysis. Acute-marked spongiosis of hair follicles in the superficial dermal layer was prominent (Figure 5C). There was a severe endothelial reaction in all of the sensitized samples with neutrophils evident inside the blood vessels. In the superficial dermis, there was a severe, acute, and diffuse inflammatory reaction comprising mainly neutrophils and eosinophils. Hyperplasia of the basal cells surrounding the sebaceous glands was evident, with little-to-no material inside the glands. The inflammatory reaction extended up to 2–3 cm deep into the skin, with patchy infiltrates containing mainly eosinophils and neutrophils, with some plasma cells and lymphocytes also evident (Figure 5D).

## 4. Discussion

This study indicated that differences amongst the cattle in BF lesion development were associated with differences in the strength of both immediate- and later-phase (24–72 h) responses to BF antigens. We also showed that there was no difference between cattle in the three lesion groups in BF numbers over the 12 months prior to hypersensitivity testing, indicating that the differences observed in the severity of lesions were not simply a reflection of the level of BF challenge.

Lesion-susceptible steers appeared to be specifically sensitive to all concentrations of BF antigens, with strong immediate reactions evident from the large wheal and flare reactions at 1 h post-injection. No significant differences between the susceptible and resistant animals were observed with either of the other antigens tested at this timepoint. Immediate hypersensitivity reactions (Type 1 response) are mediated by antigen-specific IgE, which stimulate localized mast cells to release histamine and other inflammatory mediators that cause vasodilation and plasma leakage, leading to localized edema [22]. In cattle, type 1 hypersensitivity has also been reported to other ectoparasites, including ticks (*Rhipicephalus decoloratus* and *Rhipicephalus microplus*) [19], mites (*Psoroptes ovis*) [23], and cattle grubs (*Hypoderma lineatum*) [24]. This mechanism likely underlies edema observed at BF injection sites one hour after antigen injection in lesion-susceptible steers. Edema associated with the skin reactions to the BF antigens appeared to decline after the initial reaction, but this reduction was associated with an increased skin thickness at 24, 48, and 72 h post-injection. This suggests that skinfold thickness responses were formed either as a result of a later phase of immediate hypersensitivity response, resulting from the recruitment of circulatory eosinophils and neutrophils to the challenge site [25] or due to a delayed hypersensitivity (Type 4 response) mediated by the activation of T-cell lymphocytes and the recruitment and activation of macrophages and other inflammatory cells [22].

In addition, histological examination of skin sections collected at 72 h from sites injected with 100 µg BF antigens showed necrosis of the epidermal and superficial dermal layers in the HL animals. This was likely caused by the release of the major basic proteins (MBP) from inflammatory eosinophils, and elastase enzymes from neutrophils, which can result in epidermal damage and dermal collagen disruption [26]. This further suggests that type 1 and type 4 hypersensitive responses to BF antigens are the mechanisms that instigate the development of BF lesions in susceptible cattle. These changes can lead to fibrosis in chronic wounds. Edwards et al. [14] identified atresia in a few chronic teat lesions in cattle due to fibrotic changes in response to HF feeding. The authors also reported perivascular mast cells and eosinophil infiltration with interstitial and perivascular dermatitis in some lesions, which is similar to the observations in our previous study [27].

In addition, the manifestation of clinical changes, such as the development of the serous crust and disruption of the epidermis at BF100 injection sites in the HL animals resembled the initial stages of field-observed BF lesion pathogenesis [27]. Johnson [7] reported similar clinical observations when he experimentally induced lesions in cattle by exposing them to controlled populations of BFs. Mosca et al. [16] observed similar eroded/ulcerated epidermal areas at HF feeding sites, which they suggested could be associated with the allergic response to HF feeding. Furthermore, Guglielmone et al. [15] observed dermal edema and hyperemia with the infiltration of eosinophils and neutrophils in response to HF feeding, which is also similar to our observations.

There was also a significant difference observed between the HL and NL groups in response to Md antigens at 24, 48, and 72 h post-injection, but notably no significant differences were observed in the size of the immediate response at 1 h. These later responses to Md antigens are not surprising given the relatively close phylogenetic relationship between *M. domestica* and *H. irritans* [28,29] and the likelihood of common cross-reacting antigens. House fly antigens were included in this study to assess for the possibility of cross-reactivity. Although a degree of cross-reactivity was observed, the skinfold thickness response to the BF antigens in the HL cattle was significantly greater than to the Md antigens at equivalent concentrations, indicating a BF-specific component in the response to the BF antigens. These two species have different feeding mechanisms and very likely have a different suite of salivary antigens. Buffalo flies are hematophagous and can feed up to 40 times daily [3] with a suite of anticoagulants and other enzymes to facilitate this mode of feeding [30,31]. With the high-antigen challenge presented by the high numbers of feeding BFs, it is likely that there was sensitization to BF salivary antigens in the lesion-susceptible cattle, and therefore the development of a specific type 1 response to salivary antigens was not observed with the house flies.

Although there were clear differences in the response to BF antigens between the highly susceptible animals that developed severe BF lesions and animals with no lesions, the differences between the animals with small eye lesions (LL group) and the other two groups were less marked. When animals from the HL group were compared with the LL group, differences in the response to BF antigens were found to either be significant or approach significance on a number of occasions with the reactions stronger in the HL group reflecting the stronger hypersensitive responses in the HL cattle. However, there was no significant difference observed in the size of the reactions between the LL and NL groups at any time, suggesting that the small eye lesions observed in the LL group might be the result of a different mechanism. Possible alternate explanations for the development of small eye lesions could be physical damage caused to the skin by the abrading mouthparts of BFs which rasp the skin causing minor lacerations during feeding [32], or a pruritic behavioral response to the presence of BFs.

As *Stephanofilaria* sp. nematodes are often associated with BF lesion development [7], it was originally planned to use *Stephanofilaria* antigens to determine whether the immune response to these nematodes plays a role in the development of the BF lesions. However, we were unable to source *Stephanofilaria* sp. Nematodes due to the limited access to the abattoirs and cattle herds due to COVID-19 restrictions. Due to evidence of cross-reactivity between the different species of filarial nematodes in eliciting an intradermal response [33], along with the fact that soluble antigens of dog heartworm (*Dirofilaria immitis*) have been used in the past to diagnose human filariasis caused by *Wuchereria bancrofti*, *Brugia malayi,* and *Onchocerca volvulus* [20,21], we used soluble antigens from *Onchocerca gibsoni*, a closely related nematode parasite of cattle. Although there was a significant immune response to the injection of Og antigens with wheals developing at 1 h and thickening of the skin at the nematode-injected sites, there was no significant differences observed in the response amongst the three cattle lesion groups at any time point. As all of the steers used in the current experiment tested negative for Og infection prior to antigen injections, a possible reason for the relatively large dermal responses could be the antigenic cross-reactivity of the filarial nematode with intestinal nematodes [34,35].

The lack of difference in response to the nematode antigens between the lesion groups in this study was not surprising given that the cattle used in this experiment were sourced from Southern Queensland, where *Stephanofilaria* was not found in either lesions or BFs [10]. However, recently, we reported that *Stephanofilaria* presence in the BF lesions was associated with the development of larger lesions with a greater epidermal and dermal damage, greater inflammation, and more severe adnexal destruction than in lesions where nematodes were not present [27]. The effect of the immune response to *Stephanofilaria* antigens could provide at least part of the mechanism for these effects in locations where the nematode was prevalent, but further studies with *Stephanofilaria* antigens will be necessary to confirm this. The findings from this study suggest that differences in the hypersensitivity response to BF antigens may be a key factor responsible for the differences amongst cattle in the susceptibility to BF lesion development, and further characterization of this response may aid in the identification of specific biomarkers for selecting lesion resistance in cattle.

## 5. Conclusions

The findings from this study indicate that differences in the occurrence and severity of BF-associated lesions amongst the cattle were not related to differences in the level of the BF challenge, but were determined, at least in part, by variations in the individual animal hypersensitive response to the BF antigens. Further characterization of this response may aid in the identification of specific biomarkers for breeding for lesion resistance in cattle.

## Data Availability

Not Applicable.

## Supplementary Materials

The following supporting information can be downloaded at: https://www.mdpi.com/article/10.3390/ani13122011/s1. Figure S1: Skinfold thickness response in HL and LL cattle as 24h post injection; Figure S2: Skinfold thickness response in LL and NL cattle as 24h post injection; Figure S3: Skinfold thickness response in LL and NL cattle as 48h post injection; Figure S4: Skinfold thickness response in LL and NL cattle as 72h post injection; Figure S5: Skinfold thickness response in HL and LL cattle as 48h post injection; Figure S6: Skinfold thickness response in HL and LL cattle as 72h post injection; Table S1: Skinfold thickness data (raw).
